# Supplementary material for: Identification and functional analysis of non-coding regulatory small RNA FenSr3 in Bacillus amyloliquefaciens LPB-18
Source: PeerJ. 2023 May 15;11:e15236. doi: 10.7717/peerj.15236 (PMC10194069; doi:10.7717/peerj.15236)
Supplement: Supplemental Information 4 [file peerj-11-15236-s004.zip › KO/CK-vs-T1_map/map01059.html]

KEGG PATHWAY: Biosynthesis of enediyne antibiotics - Reference pathway


|  |  |
| --- | --- |
| **Biosynthesis of enediyne antibiotics - Reference pathway** |  |

[
Pathway menu
| Organism menu
| Pathway entry
| Show description
| User data mapping
]

Enediyne natural products are potent antitumor antibiotics produced by a variety of Actinomycetes. Enediyne has a unique structure consisting of three building blocks: aromatic, sugar and enediyne core. The enediyne core contains two acetylenic groups conjugated to a double bond in a 9- or 10-membered ring, and it is synthesized by an iterative type I polyketide synthase and other tailoring proteins [MD:M00824 M00825]. The 10-membered enediyne such as calicheamicin also contains an allylic trisulfide group, which acts as a trigger for diradical formation. This diagram shows the biosynthesis of C-1027, maduropeptin, neocarzinostatin, kedarcidin and calicheamicin. Some aromatic moieties are synthesized via iterative type I polyketide synthases, and others are derived from chorismate and aromatic amino acids such as tyrosine and azatyrosine [MD:M00829 M00830 M00831 M00834 M00826 M00827 M00828 M00832]. Finally, the aromatic and sugar building blocks are attached to the enediyne core by acyltransferases, condensation enzymes and glucosyltransferases [MD:M00833].

|  |  |  |
| --- | --- | --- |
| Reference pathway | 184% 150% 122% 100% 82% 67% 55% | 图片下载 |
